# Education and Benevolence for Doctors

**Published:** 1890-05-03

**Authors:** James Paget


### Education and Benevolence for Doctors.

By Sir James Paget, Bart., F.R.S.

At the twenty-fifth annual festival, a few days ago, Sir James Paget detailed some of the grounds on which all should do their best for the promotion of the ends the Royal Medical Benevolent College at Epsom had in view. In the circular most of them had before them it was said that the objects of the institution were two-fold—educational and benevolent. Now, one's knowledge of the ordinary course of the world would seem to suggest that there was some risk in endeavouring to accomplish two such purposes through one institution, seeing that one of them appealed to the sentiment and the other to the understanding. One could certainly cite instances where the carrying out of these two together had entirely failed, but, on the other hand, too rarely, however, where the two had succeeded together. He would not say one of these objects appealed to the heart and the other to the head, but he would rather use the language of physiology, and say that there seemed to be two distinct nerve centres concerned, and they were two centres that seldom agreed in practice and were seldom balanced in the mind; rather, they seemed mutually inhibitory. He, however, ventured to say that to their great teaching institutions the rules that might be established upon other benevolent institutions absolutely failed to apply. The promotion of education and benevolence had really been the foundation of most of the great teaching institutions of this country. He need hardly refer to the older Universities, which began with a desire to educate poor students. He might allude to the schools of the great City companies, which all began as charities, and had now become among the greatest schools—the Merchant Taylors', the school at Tunbridge, St. Paul's, and he might add to them the Charterhouse, Haileybury, and others. In fact, he hardly knew one of the great schools in the country which had not had a foundation similar to this, from which it had gradually grown until it had become one of the great institutions for teaching the whole kingdom. Surely they might then feel themselves very safe if they followed an example which had proved so good; beginning with charity, never ceasing to be charitable, and arriving at excellent teaching both for the rich and the poor.

They had at Epsom College the two purposes in view—benevolent and educational—and he would consider them in that order, benevolence first. Surely it would be hard to find an institution where benevolence might be better shown than in the objects of their charity. Here were fifty members of the medical profession, all of them utterly impoverished. Surely they should help them. He supposed those who now lived in comparative prosperity could hardly realize what it is to look back, as those old men do, upon a time of fair enterprise and adventure which had ended in utter poverty. People spoke of the hardship of being sometimes driven to compulsory work. What could that be compared with the hardship of being continually forced to idleness? They could not help all the grief these old men might have suffered, but they were bound to do all they could to prevent them from having the fear of absolute poverty before their eyes, by making them feel that at least they would be maintained in their old age in quiet comfort. Then there were fifty foundation scholars. These were a number of lads born in a rank from which they must fall but for such a charity. Surely, again, these ought to be assisted. One thing more which would impress itself on many present that evening. When we looked back on our own lives there were many of us who could remember a time when it was a mere question whether we should go on in prosperity or fall into such wretchedness and poverty as hese pensioners had done; when it seemed that failure of health, if not death, a want of working power or some change in the conditions external to ourselves, would leave us in a position of destitution such as that which we were now glad to relieve in others; or that a time would come when we should have had to leave those so dear to us in such absolute poverty as they were now proud to try to assist. It could not be forgotten that there had been such moments in the lives of some of us, when if what some would call chance—but which he would rather call God's mercy—had not intervened, this would have been the fate of those left behind. Such a charity, therefore, was a just cause which they should endeavour to aid, and which they should try to get those over whom they exercised any influence to help. If he might press one more point on their notice, it was the need of banding themselves in a close, mutual, and influential brotherhood. It would not be possible for men in the medical profession not to be rivals. They must be rivals—political rivals, or scientific rivals, or rivals in some other way—and this, of course, must tend to separate them. They, however, had one bond of brotherhood, the fact that they were bound to help the weak, a bond which would dispense with all considerations, and combine them into a universal and mutual brotherhood.

Now, for the educational side. He imagined that this was in no wise separate from the purely benevolent one, for what could they do better than give these boys the best education they could, and what better help could there be for them than that they should be given an education such as the rich would be anxious for? This was really the history of all the oldest and best foundation schools in the kingdom. They began by being schools for the poor; they taught the poor so well that the rich were anxious to come amongst them, and at last the rich, seeing that there was no place for teaching to be matched by them, came to them, and had remained at them ever since. So he hoped it would be with Epsom College. They would then come to this—Epsom would match with Winchester, Charterhouse, and all those schools which were now considered the best in the kingdom. Now, respecting the method of education in the school itself, he thought it was wise, as far as he could judge of such things, that the scheme of education at Epsom College should be based upon the system which had proved so successful at Haileybury, and Marlborough. He thought that the college was wisely guided in following, in the subjects it taught, the example of the most successful colleges. They might, therefore, maintain that the college was well managed both as to its method of education and also as to the conduct of its education on the best possible plan, not only for those who were to become members of the medical profession, but for those who came there in order to obtain as good an education as could be got anywhere. One thing he noticed in the curriculum of the college, which, perhaps might not attract the attention of all of them, and that was that lately the practical teaching of natural history had been introduced. This, to his mind, was the very best course that could be adopted for the medical profession more than any other, for it brought out a boy's powers of observing for himself. He, himself, had derived great benefit from the practical study of botany, not from botany itself, but from the system of study, and the habits of observation which he thus acquired, the enthusiasm given him in the pursuit of mere facts, and the cultivation of order and brief description. Besides this he noticed that every arrangement had been made by the college for the best possible pursuit of various games and athletic exercises. Some might say that it was a most healthy thing to do, but to his mind it was more, for, together with physical health, the boys would thereby gain moral health. Here they would obtain the love of acting together, enthusiasm, and the exercise of a strong will, far more than they could in mathematics or in Greek. Here boys learned to be just to one another under mutual trial and mutual competition. He mentioned this especially, for an education in mutual honesty was important in any profession, but in none more so than the medical; for in medical practice not only was dishonesty very easy, but in no profession did it produce greater pecuniary advantage. It was, therefore, of the greatest importance that every student should be trained up to be purely honest. All this had been in the mind of the head master, in whom all reliance could be placed, and of the council with whom the management of the College rested.

